# Cancer Risk and Multiple Sclerosis: Evidence From a Large Italian Cohort

**DOI:** 10.3389/fneur.2019.00337

**Published:** 2019-04-10

**Authors:** Emanuele D'Amico, Clara G. Chisari, Sebastiano Arena, Aurora Zanghì, Simona Toscano, Salvatore Lo Fermo, Davide Maimone, Marine Castaing, Salvatore Sciacca, Mario Zappia, Francesco Patti

**Affiliations:** ^1^Section of Neurosciences, Department “GF Ingrassia”, University of Catania, Catania, Italy; ^2^Azienda Ospedaliera Garibaldi, Catania, Italy; ^3^Department of Hygiene, University of Catania, Catania, Italy

**Keywords:** multiple sclerosis, cancer risk, disease modifying therapies, immunosuppressive drugs, switching therapies

## Abstract

**Introduction:** The complexity of understanding cancer risk in MS is increased by inconsistencies in study design, and the lack of age-, sex-, and ethnicity-specific risk estimates. Aims of our study were to estimate the incidence of cancers in the MS population of Catania (Italy) and to evaluate the impact of disease-modifying treatments (DMTs) in cancer risk.

**Materials and Methods:** We screened 2,730 PwMS according to the MS criteria of Mc Donald 2010 referring to MS center of Catania in the period between 2003 and 2013. We matched database of MS patients with the Integrated Cancer of Catania-Messina-Siracusae-Enna. We calculated age and sex specific standardized incidence ratios (SIR) and the relative risk (RR) of developing cancer in MS patients treated with at least two different DMTs compared to who received one or no treatment.

**Results:** Out of 2,730, 1,180 MS patients (67.1% females; mean age 41.2 ± 12.9) were enrolled. We found 36 cancers. Global SIR was 1.18 (CI95% 0.78–1.58), with a significantly higher risk in men with a range age of 20 to 50 years [2.84; (CI95% 1.59–4.09)] and in women over 50 years [1.82 (CI95% 1.08–2.55)]. RR of developing cancer was 1.99 (CI95% 1.14–3.45) in MS patients switching one DMT and 3.38 (CI95% 1.83–6.22) in who switched at least twice.

**Discussion:** Our results demonstrated that cancer risk was not increased in our MS population; but age and sex different distribution may partly drive cancer risk. Higher cancer risk in MS patients switching more than two DMTs should take into account in treatment decision making.

## Introduction

Multiple Sclerosis (MS) is a severe acquired autoimmune neurodegenerative disease of the central nervous system (CNS) with extremely variable disease course, that usually affects persons in their third/four decades of life, even if late onset is described (1). Women had a prevalence/incidence rate approximately double than men (2, 3). In Italy, the MS prevalence ranged from 122 to 232 cases/100,000 in the mainland and Sicily, with an average of more than 109,000 MS patients in Italy (4) and an incidence of 7.0 cases /100,000 in Catania in Sicily (5).

It was suggested that MS and cancer may share some aberrant functions of immune system (6).

Indeed, in MS there is an alteration of the function of regulatory T cells (Tregs), or suppressor T cells, modulating the immunoreaction by suppressing the activation of the immune system (7). Regarding tumorigenesis, Tregs may either promote tumor growth by impeding immune surveillance, or may inhibit the growth of certain malignancies with inflammatory features (8, 9). As it is well-known that the immune system plays an important role in cancer, it is also plausible that cancer risk is modified by auto-immune diseases such as MS. It was also hypothesized that some immunological characteristics of MS disease activity could improve antitumor surveillance (10). Indeed, autoimmunity is a form of hypervigilance against self-antigens and is one of the mechanisms leading to the development of MS [11]. Following this theory, antitumor immunosurveillance should provide a physiological explanation for a reduced cancer risk in MS patients (11–14), but conflicting data exist (15, 16).

In the last 10 years, we have witnessed to great changes in the therapeutic scenario of the MS (17). Currently, available disease modifying treatments (DMTs) are able to ameliorate the course of the disease, by reducing the inflammatory activity (new clinical relapse and new radiological inflammatory lesions at brain and spine) of the immune system (18). However, the impressive results in term of controlling the disease activity by the new DMTs were accompanied by more severe safety concerns and, among them, the raised cancer incidence during the use of such drugs (teriflunomide, dimethyl fumarate, fingolimod, alemtuzumab, cladribine, and ocrelizumab) (19). In particular, cancer risk related to immunosuppressive (IS) treatments used for MS has been widely explored showing an increase cancer incidence in these patients (19, 20), but the relationship between the disease and cancer, as well as the safety profile of MS immune treatments (19, 21–24) have been not fully understood (10, 25, 26). For all these reasons, a reappraisal of cancer risk in real word MS studies (outside of the reality of clinical trials) is timely.

We aimed to compare cancer incidence among persons affected by MS to that in the general population settled in the geographic area of Catania (Italy). Moreover, we evaluated any correlation among DMTs use and cancer development.

## Materials and Methods

### Study Population

An observational retrospective analysis of prospectively collected data of patients with MS was performed at our tertiary MS center of Catania, Italy. Catania, Italy, is the second populated (1,112.328 inhabitants) and largest province of Sicily, Italy (182.90 km^2^) (www.interno.gov.it)^1^.

Patients were prospectively included using a computerized database, iMed software (iMed, Merck Serono SA; Geneva). We screened all patients who received their diagnosis between January 1st, 2003 and June 31th, 2013. Once entered the clinics, patients were followed up prospectively with at least one scheduled visit per year.

Inclusion criteria were: (a) age ≥18 years; (b) lived in the city of Catania; (c) diagnosis of MS according to the Mc Donald criteria (27); (d) at least one follow-up visit performed in the Multiple Sclerosis Center of the “Policlinico Vittorio Emanuele” of Catania in the index window.

About the DMTs use, we stratified our MS cohort according to the number of switch MS treatment (the change of treatment during to MS course due to inefficacy or safety alert):

- “no DMTs” group including patients not treated with any DMT in their MS history;
- “no switch” group including patients treated with only one DMT in their MS history;
- “1 switch” group including patients who experienced one therapeutic switch;
- “≥2 switches” group including patients who experienced at least two switches.

The study protocol was approved by the local ethics committee (Comitato Etico Catania 1) (19/2017/PO on 6th July 2017). All patients provided written informed consent. The study was conducted in accordance with the ethical principles of the Declaration of Helsinki and with the appropriate national regulations.

### Data Collection

At the time of first visit in our center, in our clinical practice for each patient, we collected the following clinical data: data of disease onset, MS clinical course, type and duration of DMTs, different switch strategy, total number of relapses, disability level assessed by Expanded Disability Status Scale (EDSS).

Oncological data regarding cancers occurred after diagnosis. We included in our analysis oncological diagnosis occurred at least 2 years after the MS diagnosis. Main groups of cancers were considered: brain and nervous system, breast, bones and joints, digestive system, endocrine system, eye and orbit, female genital system, male genital system, oral cavity and larynx, respiratory system, skin, urinary system and lymphoma-myeloma-hematopoietic or lymphatic cancer of MS were collected. We matched such data with the data of Integrated Cancer Registry of Catania-Messina-Siracusae-Enna, considering all during the observation period. We calculated age and sex specific standardized incidence ratios (SIR), as measure of the relative cancer risk.

### Outcomes

We aimed to investigate the cancer incidence among persons affected by MS to that in the general population settled in the geographic area of Catania and the relative risk (RR) of developing cancer in MS patients treated with at least two different DMTs compared to who received one or no treatment (for this analysis we included all cancer cases occurring after at least 2 years from the DMT initiation).

We also examined whether the cancer risk was different in patients with different treatment strategy: induction strategy where immunosuppressive (IS) is followed by immunomodulatory (IM) treatment, and escalation strategy where IM drugs where followed by IS treatment. Those who used interferon beta and glatiramer acetate were classified into the IM group, and those who used methotrexate, mitoxantrone, fingolimod, and azathioprine were classified into IS group. Natalizumab were included in the IS group even if its mechanism of action is not typically immunosuppressive, but because it is considered as second-line therapy.

### Statistical Analysis

Skewness and kurtosis tests were applied to the continuous variables to confirm a normal distribution and results were expressed as means and standard deviations (SD). The Mann-Whitney test was used to compare continuous and categorical variables, and Pearson's chi-square test or Fisher's exact test was used to evaluate the categorical variables.

Overall, sex-specific and age-specific estimates were reported expressed in rates per 100.000 residents associated to their relative confidence intervals, or all cancers and for main groups of cancers. These estimates were compared to those present in the Integrated Cancer Registry, available for the same period through the calculation of the rate ratios.

SIR was calculated by dividing the number of observed cases of cancer by the numbers of expected cancer cases, represented a measure of the relative risk of cancer. Wald's test assuming a Poisson distribution of the observed cases was used for determining 95% confidence interval (CI) for SIR. All statistical analysis was performed by SPSS software (Version 22; SPSS Inc., Chicago, IL, USA). Unconditional logistic regression analysis were performed using binomial “cancer yes/no” as outcome; we considered age, sex, disease duration, MS type, baseline EDSS, DMT duration (in months) and number of switches as independent factors. In order to investigate the independent effect of a risk or protective factor after adjustment for one or several other factors or to adjust for confounding variables, we carried out a multivariate model. Multivariate analysis was performed with conditional logistic regression, adjusting in the model for clinical and genetic covariables that will be significantly different to univariate analysis between cases and controls (*p*-value < 0.10). The selection of the multiple regression model will be done with the “all possible models” approach, then a regression analysis will be performed for all possible combinations. The Likelihood ratio test will be used to compare the log-likelihood of the regression model in which the aforementioned variable has been excluded. In the same way, the possible interaction (Joint effect) between two variables (“test of the violation of the proportional odds”) will be evaluated. The “test for linear trend” and the “test for departure from linear trend” will be used for quantitative variables in order to evaluate a possible dose dependent effect (“linear or trend effect”).

## Results

**Figure 1:**
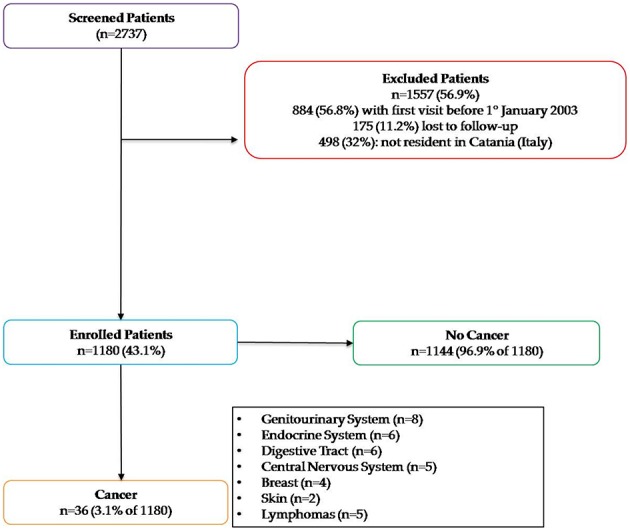
Study enrollment flowchart.

Out of 2,730 patients screened 1,180 MS (67.1% females; mean age 41.2 ± 12.9) satisfied the inclusion criteria and were enrolled for the study (Table 1, Figure 1). The mean age at MS diagnosis was 32.8 ± 17.5 years with a median observation period of 9.1 years and 8,338 person-years (2,702.2 in males and 5,636.0 in females). Demographic characteristics and clinical features of studied patients were summarized in Tables 1, 2.

**Table 1:** Clinical and demographical characteristics of the study cohort.

**Characteristics**	**Total *N* 1,180**	**Cancer *N* 36 (3.1%)**	**No cancer *N* 1,144 (96.9 %)**	***P*-value**
Females (%)	792 (67.1)	23 (63.9)	769 (67.2)	ns
Age (year) mean ± SD	41.2 ± 12.9	49.3 ± 15.6	33.1 ± 9.9	<0.001
Age at onset (year) mean ± SD	32.8 ± 17.5	37.0 ± 18.3	28.4 ± 16.6	<0.005
**FAMILY HISTORY OF MS (%)**				
No	1,153 (97.7)	35 (97.2)	1,120 (97.9)	ns
Yes	27 (2.3)	1 (2.8)	26 (2.7)	ns
**PATTERN OF DISEASE (%)**				
Relapsing-remitting	854 (72.4)	21 (58.3)	833 (72.8)	ns
Primary progressive	84 (7.1)	3 (8.3)	81 (7.1)	ns
Secondary progressive	242 (20.5)	12 (33.3)	230 (20.1)	ns
EDSS median (95%CI)	4.2 (0.0–8.5)	4.6 (1.5–7.5)	4.1 (0.0–6.5)	<0.05
N of patients reached EDSS 4.0 (%)	329 (27.9)	17 (47.2)	312 (27.3)	<0.05
Time to reach EDSS 4.0 (months) mean ± SD	52.6 ± 26.7	45.8 ± 30.5	59.4 ± 22.8	<0.05
**OCCUPATION (%)**				
Housekeeper/unemployed	446 (37.9)	15 (41.7)	431 (37.7)	ns
Student	193 (16.6)	8 (22.2)	185 (16.2)	ns
Employed	541 (45.8)	13 (36.1)	528 (45.2)	ns
**MARITAL STATUS (%)**				
Single	256 (21.7)	8 (22.2)	248 (21.7)	ns
Married	751 (63.6)	23 (63.9)	728 (63.6)	ns
Divorced/Widowed	173 (14.7)	5 (13.9)	168 (14.7)	ns
Pregnancy (%)	466 (58.8 of 792)	15 (65.2 of 23)	451 (58.6 of 769)	ns
Smoking*	349 (29.6)	15 (41.7)	334 (29.2)	ns
Alcohol use**	309 (26.2)	8 (22.2)	301 (26.3)	ns

*EDSS, Expanded disability status scale; ns, not significant; SD, standard deviation*.

* *At least 100 cigarettes (including hand rolled cigarettes, cigars, cigarillos etc.) in their lifetime and has smoked in the last 28 days*.

** *At least one glass of alcohol/die for average for at least 1 year*.

**Table 2:** Therapeutic characteristics of the cohort.

	**Total N 1,180**	**Cancer *N* 36 (3.1%)**	**No cancer N 1,144 (96.9 %)**	***P*-value**
No DMT (%)	113 (9.6)	1 (2.8)	112 (9.8)	ns
No switch (%)	309 (26.2)	5 (13.9)	304(26.6)	ns
1 switch (%)	501 (42.5)	8 (22.2)	493 (43.1)	<0.01
>2 switches (%)	257 (21.8)	22 (61.1)	235 (20.5)	<0.001
N. Treated patients (%)	1067 (90.4)	35 (97.2)	1032 (90.2)	ns
Interferon beta (%)	436 (40.8)	12 (34.3)	424 (41.1)	ns
Persons/year	2724.3	106.5	3871.8	ns
Glatiramer acetate (%)	268 (25.1)	10 (28.6)	258 (25)	ns
Person/year	1964.8	99.6	1745.1	ns
Fingolimod (%)	118 (11.1)	5 (14.3)	113 (10.9)	ns
Person/year	845.7	49.6	743.8	ns
Natalizumab (%)	142 (13.3)	0	142 (13.8)	ns
Person/year	1056.9		1056.9	ns
Azathioprine (%)	53 (5.0)	4 (11.4)	49 (4.7)	ns
Person/year	436.1	38.7	401.1	ns
Mitoxantrone (%)	16 (1.5)	2 (5.7)	14 (1.4)	ns
Person/year	144.2	16.4	128.4	
Methotrexate (%)	34 (3.2)	2 (5.7)	32 (2.8)	ns
Person/year	298.7	17.2	274.3	
DMT duration (months) mean ± SD	86.4 ± 15.9	86.4 ± 21.6	80.6 ± 17.3	ns
Escalation strategy (%)	389 (33)	6 (16.6)	383 (33.5)	ns
Induction strategy (%)	112 (9.5)	2 (5.6)	110 (9.6)	ns

“*No DMTs” group including patients not treated with any DMT in their MS history; “no switch” group including patients treated with only one DMT in their MS history; “1 switch“ group including patients who experienced on therapeutic switch; “>2 switches “group including patients who experienced at least two switches. DMT, disease modifying therapies; ns, not significant; SD, standard deviation; Escalation strategy, sequence of switching therapies with immunomodulatory (IM) followed by immunosuppressive (IS) drugs; Induction strategy, sequence of switching therapies with IS followed by IM drugs*.

We found 36 cancers in 36 patients (3.1% of enrolled population). Global SIR of 1.18 (CI 95% 0.78–1.58) was obtained. Stratifying for age and sex, we found a significantly higher risk in men with the range age 20–50 years (2.84; CI 95% 1.59–4.09) and in women over 50 years (1.82; CI 95% 1.08–2.55) (Table 3).

**Table 3:** Standardized incidence ratios.

**Range of age**	**Total**	**SIR (CI 95%)**	
		**Males**	**Females**
Total	(*N* = 36) 1.18 (0.78–1.58)	(*N* = 13) 0.81 (0.0–2.06)	(*N* = 23) 0.89 (0.0–2.19)
20–50	(*N* = 15) 1.12 (0.54–1.69)	(*N* = 9) 2.84 (1.59–4.09)^*^	(*N* = 7) 65 (0.0–1.30)
>50	(*N* = 21) 1.24 (0.68–1.80)	(*N* = 4) 4.0 (0.0–1.29)	(*N* = 16) 1.82 (1.08–2.55)^*^

* *SIR (Standardized Incidence Ratio) statistically significant (p < 0.05)*.

About the 36 cancers, 8 (22.2%) were primary located in the genitourinary system (all bladder cancers), 6 (16.7%) thyroid cancers, 6 (16.7%) were colon cancers, 5 (13.9%) were of the CNS, 5 (13.9%) were lymphomas, 4 (11.1%) were breast cancers and 2 (5.6%) were melanomas. Further analyses with respect to the cancer sites in MS patients resulted in an increased risk of genitourinary cancer [SIR = 1.65 (95% CI, 1.34–2.35)], and for thyroid cancer [SIR = 1.32 (95% CI, 1.03–2.49)], but not for breast cancers [SIR = 1.17 (95% CI, 0.98–2.42)] and cancers which are located in nervous system [SIR = 1.03 (95% CI, 1.01–5.05)].

Patients with cancer were older than others (49.3 ± 15.6 vs. 33.1. ± 9.9, *p* < 0.001), with higher age at MS onset (37.0 ± 18.3 vs. 28.4 ± 16.6, *p* < 0.005). Moreover, MS patients with cancer had longer MS disease duration, worse disability (higher mean EDSS), that is an increased percentage of patients who got EDSS 4.0 (47.2 vs. 27.3%, *p* < 0.05) which means a limitation in ambulation (Tables 1, 2).

About DMTs use, the RR of developing cancer in “no DMTs” group was 1.03 CI 95% (0.56–1.41), in “no switch” group was 1.17 CI 95% (1.02–2.34), 1.99 (CI 95% 1.14–3.45) in MS patients who switched one DMT and 3.38 (CI 95% 1.83–6.22) in MS patients who switched at least twice.

The multivariate analysis showed that age, age at onset, disease duration, and more that 2 switches were associated with a higher risk of developing cancer (Table 4).

**Table 4:** Multivariate analysis.

	**Univariate**			**Multivariate**		
	**OR**	**95% CI**	***P*-value**	**OR**	**95% CI**	***P*-value**
Age	1.98	1.13–13.29	<0.01	1.31	1.10–5.12	<0.05
**Sex**						
Male	1.59	0.81–9.19	ns	1.23	0.87–8.12	ns
Female	1.46	0.89–8.71	ns	1.46	0.91–7.49	ns
Age at onset	3.65	1.51–8.63	<0.01	2.17	1.16–12.69	<0.01
Smoking	3.11	1.01–12.6	<0.05	2.59	0.94–16.3	ns
Alcohol use	1.36	0.94–6.35	ns	1.13	0.91–6.29	ns
Disease duration	2.01	1.08–8.47	<0.05	2.24	1.56–6.13	<0.05
DMT duration (months)	1.32	0.95–6.68	ns	1.29	0.81–7.03	ns
Escalation strategy	1.41	1.03–12.3	<0.05	1.54	0.94–19.60	ns
Induction strategy	2.05	1.09–5.67	ns	1.41	0.91–4.84	ns
No DMT	0.86	0.71–2.21	ns	0.90	0.75–3.03	ns
No switch	0.89	0.64–2.09	ns	0.91	0.81–5.21	ns
1 switch	1.30	1.23–9.64	<0.05	1.43	0.96–11.3	ns
>2 switches	2.09	1.09–7.54	<0.01	1.78	1.19–6.12	<0.05

“*No DMTs” group including patients not treated with any DMT in their MS history; “no switch” group including patients treated with only one DMT in their MS history; “1 switch“ group including patients who experienced on therapeutic switch; “>2 switches “group including patients who experienced at least two switches. DMT, disease modifying therapies; ns, not significant; SD, standard deviation; Escalation strategy, sequence of switching therapies with immunomodulatory (IM) followed by immunosuppressive (IS) drugs; Induction strategy, sequence of switching therapies with IS followed by IM drugs*.

## Discussion

Our results showed that incidence of cancer in our MS cohort was not higher than general population. However, we found a significantly higher incidence of cancer in men in the range age 20–50 years and in women over 50 years. Our MS patients who experienced at least two therapeutic switches DMTs had a higher risk for the development of cancers. Moreover, in our multivariate analysis, age, disease duration and more than 2 switches were associated with a higher cancer risk. As described above, data about MS and cancer risk are conflicting with a majority of these evidences suggesting that patients with MS are at reduced or at least not overall increased risk for the development of cancer (10, 16, 26, 28).

Our finding of a higher risk in younger men and older women in our MS population deserves attention. For most MS cohorts reported in the literature, there is no relevant difference in cancer risk between men and women (10, 24, 29, 30). It has been widely demonstrated that aging is able to drive degenerative diseases and hyperplastic pathology; indeed, it has been demonstrated that senescent cells are able to promote the development of a tissue microenvironment that is permissive for the cancer initiation and progression (31). Thus, we could speculate that any increase in cancer prevalence in such cohorts (men 20–50 and women over 50) could be influenced by several factors, such as hormonal change, that may drive cancer risk in these subpopulations (32). The gender difference in cancer sensibility is a consistent finding in cancer epidemiology studies. The expression of X-linked genes and sex steroid hormones interacting with specific receptors, may influence the immunological response to several stimuli, possibly resulting in the different male/female cancer risk incidence ratio (33). Indeed, the higher incidence of cancers in women over 50 years, may support these data, as the estrogens levels are typically reduced in this age range.

About cancer site, our data are in agree with the current literature, showing that, although the overall risk for malignancies in the MS patients does not seem to be increased, a possible raising of certain cancers such as breast cancer (11, 15) cancers of the CNS (15, 34), the urinary tract system and nasopharynx (15) was described. To better study such phenomenon, we should understand the pharmacokinetic of the administered drugs. In fact, the majority of molecules used for MS treatment have the ability to be retained (in the form of various metabolites) for extremely variable time in certain tissues (35). Indeed, about the genitourinary tract, some Authors found not significantly increase in bladder cancer risk (25). However, stratifying for sex and age, females patients with MS at the ages of 30 to 39 years and female patients with MS for more than 10 years exhibited an increased risk of bladder cancer, whereas in men the risk of bladder cancer was increased 1 to 9 years after MS diagnosis (25, 26).

An interesting data is the higher incidence of thyroid cancer in our cohort, not confirmed by other reports (36, 37). It has been well-described that thyroid cancer incidence is increased in volcanic area such as Catania (38, 39). However, this data could be explained by the immune-mediated inflammation of this organ that is a common comorbidity with MS (40).

A small but significantly increased risk of breast cancer in patients with MS was reported in a few studies; in detail a 1.6-fold (RR = 1.56) increased risk of breast cancer was found among a cohort of 11,817 MS patients (25, 41). The higher report of CNS tumors in the literature could be due to a misclassification of benign MS lesions, as explained in other studies (10, 42). Moreover, some Authors suggested that a potential surveillance bias could lead to more cases of meningioma in MS patients compared to the controls (43).

In our cohort, we demonstrated that the risk of cancer in patients who had received only one DMT was not increased, in line with other studies (22, 42, 44). A recent Italian study demonstrated that cancer risk was higher in MS patients with previous IS exposure compared with patients not exposed to IS, matched for age. In this study the risk of cancer in MS patients exposed to IS was related to the duration of exposure and the cumulative dose, but not to a specific IS (24). Conversely, we did not reply this result, but we found a higher cancer risk in MS patients switching more than two DMTs. This latter is in line with another study showing that RR of cancer in patients treated with only one immunomodulating drug was not increased, but it raised in patients treated with more than three immunomodulating drugs and/or IS (44). Thus, we may hypothesize that MS patients experiencing therapeutic failure with different immunomodulating and/or IS could have an enhanced risk of cancer because to being exposed to different molecules with different mechanisms of action may negatively influence the innate and adaptive immune systems and make these patients more sensitive of carcinogenesis (19, 26, 45).

Finally, we observed that MS patients with cancer diagnosis had a worse EDSS and got EDSS 4.0 faster compared to other MS patients. Indeed, it has been well-demonstrated that MS course depend not only on disease characteristics but also on several factors including comorbidities, as cancer (20, 46, 47). It has been demonstrated that mortality is increased in MS with psychiatric, cerebrovascular, cardiovascular, lung, diabetes, cancer, or Parkinson disease comorbidities (48). However, the impact of cancer diagnosis on the course of MS, as well as on life expectancy and of quality of life of MS patients, are not fully understood (46).

Our multivariate analysis showed that age, age at onset, disease duration and >2 switches, are associated with a higher cancer risk (Table 4). As for aging, it is also known that higher age at onset may expose patients to more frequent comorbidities (49) and, thus, to higher cancer risk as well. More interestingly, the higher risk in those patients switching twice may suggest that the mechanisms by which DMTs influence the immune system of MS patients are not fully understood and this may significantly impact the MS management leading to a more careful evaluation of the reasons to switching therapy.

Our study had several limitations. Firstly, the restricted area in which it was conducted may lead to a number of cancer cases that is too small to obtain conclusions for each cancer subtype. Moreover, we cannot exclude also the underestimation of the number of cancers due to the possible absence of follow-up or non-registered cancers. Secondly, we did not take into account data about the duration of DMTs exposure, as the cancer risk could be time-dependent. In addition, the intensity and duration of exposure of other factor as alcohol consumption, tobacco use, sun exposure, hormone therapy etc., need to be evaluated in the next studies, as the impact of dose is well-known for carcinogenesis. Moreover, we carried out our study in the period between 2003–2013, when dimethyl fumarate, teriflunomide, cladribine, alemtuzumab, and ocrelizumab were not available yet; hence, this could prevent us to understand the possible impact of these new drugs on carcinogenesis. A recent review showed that for dimethyl fumarate and teriflunomide an increased cancer risk was not demonstrated, whilst the use of alemtuzumab and cladribine require caution because of their potential risk of developing malignancies (19). However, real experience studies are needed in order to assess the cancer risk in patients treated with these molecules. Finally, other risk factors should be also explored in future studies (estrogen use, UV exposure, nutritional habits, body mass index, viral infections, etc).

In conclusion, our results suggest that some MS subpopulations may be more susceptible to the risk of develop cancer. The relationship between MS and risk of cancer is complex- Although the immune-mediated pathogenesis of MS enhancing the immune surveillance due to activation of inflammatory cells (23), may “protect” from carcinogenesis, on the other hand, current studies indicate that MS is largely a heterogeneous disease process, which involves a dysregulation of both innate and adaptive immune-mediated inflammatory mechanisms that ultimately contribute to demyelination and neurodegeneration (50). Thus, we could speculate that the dysregulation of the immune system typically responsible for the pathogenesis of MS disease, and the immunological consequences of using drugs with different mechanisms of action may enhance cancer risk.

Furthermore, the finding of higher cancers risk in patients who switched at least twice may have significant implications on MS management, influencing treatment decision making process. Indeed, our results raised the question of long-term toxicity of MS drugs, highlighting the importance of taking into account the consequences of a succession exposure to many different molecules, in particular with the development of new MS therapies more and more powerful.

More prospective researches are needed via epidemiology and experimental study designs to confirm these data.

## Ethics Statement

This study was carried out in accordance with the recommendations of the Ethics Committee of Catania (Catania 1) with written informed consent from all subjects. All subjects gave written informed consent in accordance with the Declaration of Helsinki. The protocol was approved by the Ethics Committee of Catania (Catania 1) (No. 19/2017/PO).

## Author's Note

The results described in this paper were partially presented as poster presentation at ECTRIMS meeting, 25th−28th October 2017 Paris, France.

## Author Contributions

ED and CC contributed to data curation, and drafting, reviewing and editing the manuscript. SA contributed to data curation and investigation. AZ, ST, SLF, and DM contributed to data curation. MC contributed to data curation, methodology and formal analysis. SS contributed to data curation and formal analysis. MZ performed conceptualization, supervision and validation. FP contributed to the methodology, supervision, and reviewing, editing and validation of the manuscript.

### Conflict of Interest Statement

ED received personal fees by Biogen and Sanofi Genzyme. He also received travel funding from Bayer, Biogen, TEVA, Sanofi Genzyme and Merck. CC received personal fees by Sanofi Genzyme. She also received travel funding from Bayer, TEVA, Sanofi Genzyme, Almirall, Novartis, Roche, Biogen, and Merck. AZ received travel funding from Bayer and Sanofi Genzyme. ST received travel funding from Biogen. SL received travel funding from from Bayer, TEVA, Sanofi Genzyme, Novartis, Biogen, and Merck. DM served on the advisory board for Bayer, Biogen, Merck, Novartis, Roche, Sanofi, TEVA, and also received personal fees for speaking activities at congresses or sponsored symposia. MZ has received honoraria for speaking activities by Bayer Schering, Biogen, Merck, Novartis, TEVA and Sanofi Aventis; he also served as advisory board member the following companies: Bayer Schering, Biogen, Merck, Novartis; he received grant for congress participation from Bayer Schering, Biogen Idec, Merck, Novartis, Sanofi Aventis and TEVA. FP has received honoraria for speaking activities by Bayer Schering, Biogen, Merck, Novartis, Roche, TEVA and Sanofi Aventis; he also served as advisory board member the following companies: Bayer Schering, Roche, Biogen, Merck, Novartis; he was also funded by Pfeizer and FISM for epidemiological studies; finally he received grant for congress participation from Bayer Schering, Roche, Biogen, Merck, Novartis, Sanofi Aventis, and TEVA. The remaining authors declare that the research was conducted in the absence of any commercial or financial relationships that could be construed as a potential conflict of interest.

## Footnotes

^1^Censis Report. *42*° *Rapporto Censis sulla Società Italiana*. (2008). Available online at: www.interno.gov.it (accessed April 27, 2017).
